# CmeABC Multidrug Efflux Pump Contributes to Antibiotic Resistance and Promotes Campylobacter jejuni Survival and Multiplication in Acanthamoeba polyphaga

**DOI:** 10.1128/AEM.01600-17

**Published:** 2017-10-31

**Authors:** Ana Vieira, Amritha Ramesh, Alan M. Seddon, Andrey V. Karlyshev

**Affiliations:** Faculty of Science, Engineering, and Computing, Kingston University, Kingston upon Thames, Surrey, United Kingdom; University of Manchester

**Keywords:** Campylobacter jejuni, Acanthamoeba polyphaga, host cell invasion, survival and multiplication, antibiotic resistance, multidrug efflux pumps, biofilm formation, motility, CmeB

## Abstract

Campylobacter jejuni is a foodborne pathogen that is recognized as the leading cause of human bacterial gastroenteritis. The widespread use of antibiotics in medicine and in animal husbandry has led to an increased incidence of antibiotic resistance in Campylobacter. In addition to a role in multidrug resistance (MDR), the Campylobacter CmeABC resistance-nodulation-division (RND)-type efflux pump may be involved in virulence. As a vehicle for pathogenic microorganisms, the protozoan Acanthamoeba is a good model for investigations of bacterial survival in the environment and the molecular mechanisms of pathogenicity. The interaction between C. jejuni 81-176 and Acanthamoeba polyphaga was investigated in this study by using a modified gentamicin protection assay. In addition, a possible role for the CmeABC MDR pump in this interaction was explored. Here we report that this MDR pump is beneficial for the intracellular survival and multiplication of C. jejuni in A. polyphaga but is dispensable for biofilm formation and motility.

**IMPORTANCE** The endosymbiotic relationship between amoebae and microbial pathogens may contribute to persistence and spreading of the latter in the environment, which has significant implications for human health. In this study, we found that Campylobacter jejuni was able to survive and to multiply inside Acanthamoeba polyphaga; since these microorganisms can coexist in the same environment (e.g., on poultry farms), the latter may increase the risk of infection with Campylobacter. Our data suggest that, in addition to its role in antibiotic resistance, the CmeABC MDR efflux pump plays a role in bacterial survival within amoebae. Furthermore, we demonstrated synergistic effects of the CmeABC MDR efflux pump and TetO on bacterial resistance to tetracycline. Due to its role in both the antibiotic resistance and the virulence of C. jejuni, the CmeABC MDR efflux pump could be considered a good target for the development of antibacterial drugs against this pathogen.

## INTRODUCTION

Campylobacter jejuni is a microaerophilic, spiral-shaped, Gram-negative, motile, foodborne pathogen that is recognized as the main cause of bacterial gastroenteritis worldwide (1). Most commonly, the disease is associated with consumption of undercooked poultry or contaminated water, where Campylobacter can coexist with protozoa and form biofilms (2). Antimicrobial therapy is warranted for immunocompromised patients or patients with severe infections and, although most people recover quickly from this disease, others may develop rare neurodegenerative disorders such as Guillain-Barré syndrome (GBS), which manifests as paralysis, requiring extensive medical treatment (3).

The ability of C. jejuni to invade host cells is important for pathogenicity (4). To establish infection in humans, C. jejuni invades the gut epithelial layer and colonizes the intestine by employing a variety of virulence factors (2). The CmeABC multidrug resistance (MDR) efflux pump plays a key role in C. jejuni colonization of chickens (a natural host and a major reservoir of Campylobacter) by mediating resistance to bile salts present in the intestinal tract (5). The MDR pump is a tripartite efflux system belonging to the resistance-nodulation-division (RND) superfamily of bacterial transporters. It consists of three components, i.e., the outer membrane channel-forming protein CmeC, the inner membrane drug transporter CmeB, and the periplasmic protein CmeA, which bridges CmeB and CmeC (6). The CmeABC complex contributes to the intrinsic resistance of C. jejuni to a broad range of antibiotics, heavy metals, and other antimicrobial agents (7). There are high levels of variation in the amino acid sequences of the CmeB protein among C. jejuni strains, which may have an impact on the function of this transporter (8). Investigation of the molecular mechanisms of antibiotic resistance is important for control of the dissemination of multidrug-resistant bacteria (9).

Although most studies of MDR pumps have focused on investigations of their role as antibiotic resistance determinants, MDR pumps may also play a role in bacterial pathogenesis (10–15). For example, the CmeB homologues AcrB (Salmonella enterica and Klebsiella pneumoniae) and MexB (Pseudomonas aeruginosa) are required for invasion of host cells and virulence (16–19), suggesting a possible contribution of the CmeB protein to the pathogenic properties of C. jejuni.

The CmeABC efflux pump of C. jejuni is known to be required for resistance to antibiotics, bile salts, and some disinfectants, as well for host colonization (5, 7, 20). However, its role in biofilm formation, motility, or survival within amoebae has not yet been studied.

Acanthamoeba is a genus of amoebae containing free-living protist pathogens that are widely spread in water environments (21, 22). These eukaryotic organisms are characterized by spine-like structures on their surface, which allows them to adhere, to move, and to capture their prey by phagocytosis (21). During their life cycle, acanthamoebae can adopt two reversible forms, i.e., trophozoite cells, which are able to feed on microbes, and dormant, double-walled, cyst cells with minimal metabolic activity, which are formed under adverse environmental conditions such as extreme temperatures or pH values (22). Acanthamoebae are opportunistic pathogens that are capable of causing serious human infections, including blinding keratitis, which is associated mostly with contact lens users, and fatal granulomatous encephalitis, which occurs mainly in immunocompromised patients (21).

An increasing number of microorganisms, such as bacteria and viruses, have been reported to benefit from interactions with these free-living pathogens, as the amoebae play a role as a reservoir, allowing the microorganisms to escape predation and perhaps enabling them to survive and/or to multiply inside their hosts and to be transmitted in the environment (23). Pathogenic microorganisms residing inside amoebae become more virulent and less susceptible to antibiotics and disinfectants, making it difficult to eradicate them from public water supplies and creating problems for human and animal health (24–26).

Amoebae are easy to handle experimentally and represent an attractive and simple model of infection to study host-pathogen interactions *in vitro*, allowing the discovery of new bacterial virulence factors, which may facilitate the development of new antibacterial therapeutic agents (27). Also, amoebae exhibit features similar to those of macrophages, especially in the way they capture their prey by phagocytosis (28).

Elucidation of the molecular mechanisms involved in these interactions is relevant to public health, since microbial pathogens and acanthamoebae can coexist in the same environments, e.g., in the water of industrial poultry houses (despite stringent biosecurity measures) (29). Such associations may be beneficial to the acanthamoebae and/or the bacteria, which may lead to symbiotic relationships between these microorganisms (30).

The molecular mechanisms of interactions between C. jejuni and these eukaryotic hosts are not well understood. The results from a few publications describing the interactions between Acanthamoeba polyphaga and C. jejuni are contradictory (31). While some studies suggest the ability of C. jejuni to survive and/or to multiply within amoebae (32–39), others support an extracellular mode of survival only (40–43). These conflicting results may be explained by variations in the strains of C. jejuni and amoebae and the use of different methods, such as whether the *in vitro* survival experiments involved gentamicin treatment.

The objectives of this study were (i) to elucidate the type of interactions between C. jejuni and A. polyphaga, (ii) to investigate whether the CmeB multidrug efflux transporter is involved in this interaction, (iii) to explore other possible biological functions for this transporter in C. jejuni, and (iv) to investigate the contribution of variations in the amino acid sequence encoded by *cmeB* to the tetracycline resistance of C. jejuni. To accomplish the first two aims, the standard gentamicin protection method was modified to ensure more efficient elimination of both attached and extracellular bacteria (which could escape to the medium and cause reinfection, thus potentially producing misleading data).

The association between foodborne pathogens and protozoa leads to serious consequences for food safety, increasing the risk of infection (44). Deciphering the molecular mechanisms of Campylobacter-amoeba interactions will facilitate a better understanding of the lifestyle of this foodborne pathogen, aiding in the development of novel intervention strategies.

## RESULTS

### C. jejuni 81-176 is able to survive and to multiply within A. polyphaga.

The survival of C. jejuni at different temperatures was investigated *in vitro* by using the A. polyphaga model of infection. Due to its lifestyle, Campylobacter is likely to encounter a wide range of environmental conditions, including different temperatures. In order to survive, it must be able to sense, to adapt to, and to respond to temperature fluctuations (45). The temperatures 25°C and 37°C were selected for this study, to mimic environmental and human host temperatures, respectively.

**FIG 1:**
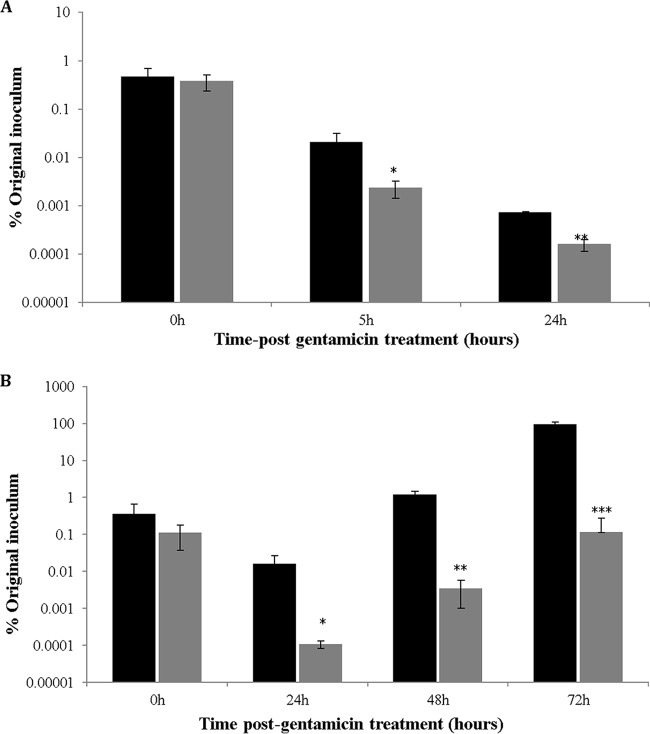
C. jejuni 81-176 is able to survive and to multiply inside A. polyphaga. (A) Intracellular survival was determined by CFU counting at 0, 5, and 24 h after gentamicin treatment at 25°C, under aerobic conditions. (B) Intracellular multiplication was determined at 0, 24, 48, and 72 h after gentamicin treatment at 37°C, under aerobic conditions. Black bars represent bacterial counts obtained with the standard gentamicin protection assay, and gray bars represent bacterial counts obtained with a modified version developed in this study. *P* values, referring to comparisons between the samples at each time point, were as follows: panel A: 0 h, *P* = 0.583; 5 h, *P* = 0.031; 24 h, *P* = 0.00003; panel B: 0 h, *P* = 0.166; 24 h, *P* = 0.03; 48 h, *P* = 0.001; 72 h, *P* = 0.00028. *, 0.01 < *P* ≤ 0.05; **, 0.001 < *P* ≤ 0.01; ***, *P* ≤ 0.001.

To elucidate the interaction between C. jejuni 81-176 and A. polyphaga, the standard gentamicin protection method (19) was modified by adding an extra 1 h of gentamicin treatment at the later incubation time points, before lysis of the cells; this avoided quantification of bacteria that were attached or had escaped to the extracellular medium and that were potentially capable of reinvasion, since the gentamicin concentration used (100 μg/ml) was sufficient to kill C. jejuni wild-type (WT) and mutant strains (data not shown). By using the modified version of this method, we observed a substantial decrease in the numbers of intracellular bacteria, compared with the standard version, at both 25°C (Fig. 1A) and 37°C (Fig. 1B).

At 25°C and 0 h (defined as the time point immediately after the first gentamicin treatment), the methods produced similar CFU counts (Fig. 1A). However, much greater decreases in intracellular bacteria were observed at 5 h and 24 h with the modified procedure (Fig. 1A). Since no colonies were detectable at this temperature after 48 h (data not shown), no further time points are shown. These data indicate that strain 81-176 can invade and survive inside amoebae at 25°C for a certain period.

Because the optimal growth temperature for C. jejuni is 37°C, time points for this temperature were extended to 72 h. At this temperature, the decrease in the intracellular bacterial numbers at 24 h after gentamicin treatment with the modified gentamicin method was even more pronounced, and even greater differences were observed at the later time points (Fig. 1B). With both methods, initial reductions in the CFU counts were followed by increases after prolonged incubation, suggesting bacterial multiplication (Fig. 1B). Although the methods demonstrated the same trends in changes in CFU counts with time, the modified version allowed more accurate quantification of only intracellular bacteria and was used in all subsequent experiments.

A different modification of the gentamicin protection method, in which a lower concentration of gentamicin is constantly maintained, was described elsewhere (46). Using that method, however, we observed that C. jejuni 81-176 was not able to multiply intracellularly and was not detected 48 h postinfection (data not shown), probably because the antibiotic was able to enter the amoebic cells during prolonged incubations. This observation is in accordance with previous studies reporting the ability of gentamicin to enter host cells during prolonged incubations and to kill intracellular bacteria (47, 48).

The numbers of viable extracellular bacteria were monitored for a 6-day period. At 96 h postinfection, a significant increase in bacterial counts was observed, compared with bacteria incubated in medium alone. Moreover, after 6 days of incubation, the presence of amoebae allowed the isolation of viable bacteria, while none could be detected in the absence of amoebae (see Fig. S1 in the supplemental material). In summary, these data support both extracellular and intracellular modes of survival for C. jejuni 81-176 in cocultures with A. polyphaga at different temperatures.

### CmeB contributes to C. jejuni 81-176 intracellular survival and multiplication within A. polyphaga.

To check whether the C. jejuni CmeB transporter is required for survival and multiplication within amoebae, the *cmeB* gene of strain 81-176 was inactivated by insertional mutagenesis (49) to create the 81-176/*cmeB*::*kan*^*r*^ mutant. Complementation was achieved by replacement of the mutated gene with its wild-type copy and selection of the derivative at a tetracycline concentration that did not support the growth of the mutant strain (Table 1). Construction of the mutant strain and its complementation derivative was confirmed by PCR (Fig. S2A). The mutation had no impact on the bacterial growth rate (Fig. S2B).

**TABLE 1:** Susceptibility of C. jejuni WT strains and their *cmeB* mutants to tetracycline

Campylobacter jejuni strain	Tetracycline MIC (mean ± SD) (μg/μl)^*a*^	Fold change (WT strain vs mutant strain)
81-176/*cmeB*^+^/pTet^+^ (WT)	62.5 ± 0.010	
81-176/*cmeB*^−^/pTet^+^	7.8 ± 0.020	8
11168H/*cmeB*^+^/pTet^−^ (WT)	0.12 ± 0.017	
11168/*cmeB*^−^/pTet^−^	0.06 ± 0.017	2
G1/*cmeB*^+^/pTet^−^ (WT)	1.95 ± 0.002	
G1/*cmeB*^−^/pTet^−^	0.06 ± 0.011	32.5
G1/*cmeB*^+^/pTet^+^	500 ± 0.031	256

a MICs for tetracycline were determined by the microdilution method in MH-F broth, according to the EUCAST recommendations (64). Tetracycline MICs were determined for strains 81-176, 11168H, and G1 and their respective *cmeB* mutants; three clonal isolates were tested for each strain. The tetracycline MIC value was also determined for a derivative of the G1 strain containing the pTet plasmid from C. jejuni strain 81-176.

The tissue culture experiments involved lysis of the amoebic cells with 0.1% Triton X-100 for 15 min at room temperature. Because the 81-176 *cmeB* mutant strain was shown to be susceptible to this detergent (5), experiments were performed to ensure that the data obtained were genuine and not experimental artifacts. The effect of 0.1% (vol/vol) Triton X-100 was investigated by simulating its use in cell culture experiments in which this detergent was added to the bacteria for a short period. Under those conditions, CFU counts for the 81-176*/cmeB*::*kan*^*r*^ mutant were similar to those of the WT and complemented strains (Fig. S3), suggesting that Triton X-100 had no detrimental effect on bacterial viability.

**FIG 2:**
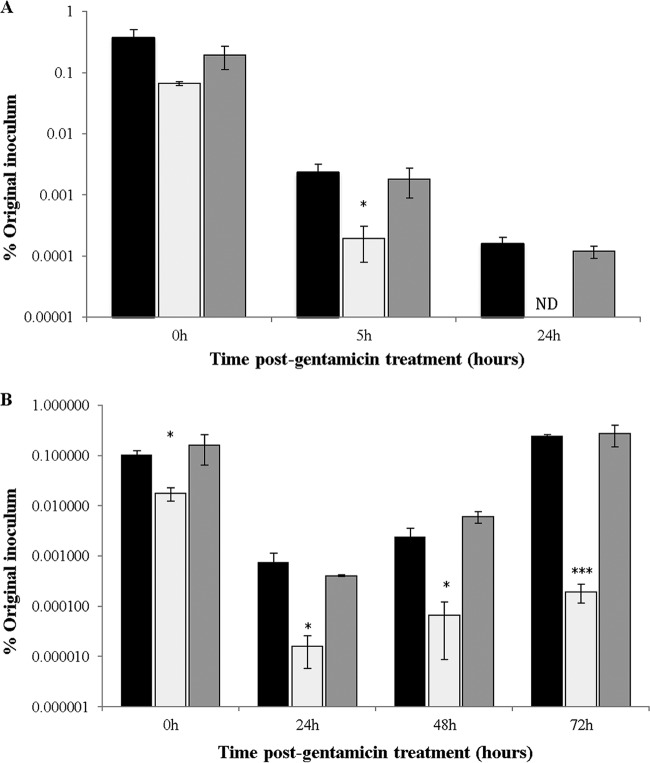
CmeB is required for survival and multiplication of C. jejuni 81-176 in A. polyphaga. (A) Intracellular survival was determined by CFU counting at 0, 5, and 24 h after gentamicin treatment at 25°C, under aerobic conditions. (B) Intracellular multiplication was determined at 0, 24, 48, and 72 h after gentamicin treatment at 37°C, under aerobic conditions. Black bars, 81-176; white bars, 81-176/*cmeB*::*kan*^*r*^ mutant; gray bars, 81-176/*cmeB*::*kan*^*r*^/*cmeB* complementation derivative. *P* values, referring to comparisons between the values for WT and mutant strains at each time point, were as follows: panel A: 0 h, *P* = 0.018; 5 h, *P* = 0.015; panel B: 0 h, *P* = 0.021; 24 h, *P* = 0.031; 48 h, *P* = 0.028; 72 h, *P* = 0.000004. *, 0.01 < *P* ≤ 0.05; ***, *P* ≤ 0.001. ND, not detected.

At both 25°C and 37°C, the numbers of intracellular bacteria for the 81-176*/cmeB*::*kan*^*r*^ mutant were significantly lower than those of the wild-type strain, with the difference increasing after longer incubations (Fig. 2A). In contrast to the WT strain, no amplification of bacteria was observed at 37°C even after 72 h (Fig. 2B). There was no statistically valid difference in CFU numbers for the mutant at 48 h and 72 h, compared to those at 24 h (*P* = 0.24 and *P* = 0.09, respectively). Complementation of the 81-176 *cmeB* mutant strain restored the phenotype in all experiments. In summary, the results suggest that the C. jejuni 81-176 CmeB is required for bacterial survival and multiplication in acanthamoebae.

### CmeB is not required for biofilm formation and motility of C. jejuni 81-176.

**FIG 3:**
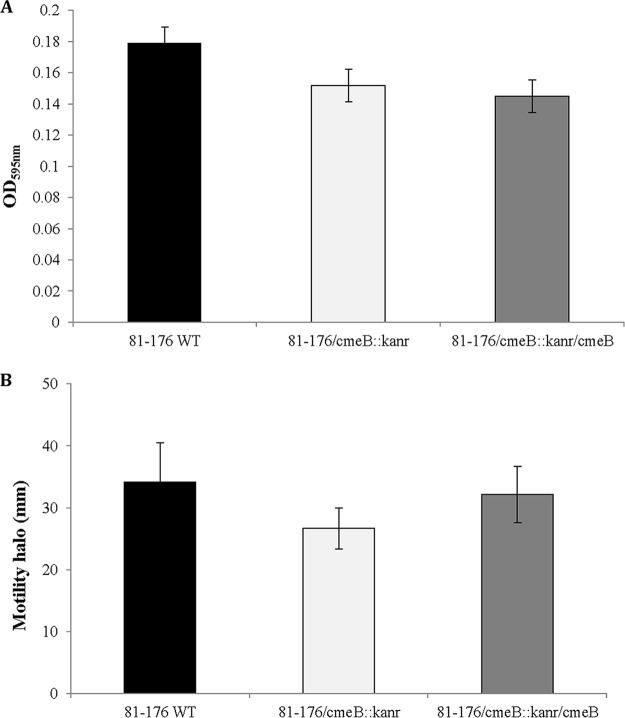
The *cmeB* mutation does not affect biofilm formation and motility. (A) Quantification of biofilms at the air-liquid interface of the glass tubes. The absorbance values measured for the WT, *cmeB* mutant, and complemented strains were 0.179 ± 0.06, 0.151 ± 0.01, and 0.145 ± 0.02, respectively. No statistically significant difference (*P* = 0.443) in biofilm quantities between the WT and *cmeB* mutant strains was observed. (B) Quantification of growth zones in BHI soft-agar motility plates inoculated with different C. jejuni strains. The average diameters of bacterial growth for the WT, *cmeB* mutant, and complemented strains were 34.2 ± 6.37 mm, 26.7 ± 3.33 mm, and 32.2 ± 4.54 mm, respectively. No statistically significant difference in growth zones (*P* = 0.15) between the WT and *cmeB* mutant strains was observed. Black bars, 81-176; white bars, 81-176/*cmeB*::*kan*^*r*^ mutant; gray bars, 81-176/*cmeB*::*kan*^*r*^/*cmeB* complementation derivative.

The ability to form biofilms is an important factor in the lifestyle of C. jejuni (50). Some studies showed that inactivation of the multidrug efflux pumps could prevent biofilm formation (51). Using a previously described assay to study pellicle formation at the air-liquid interface (52), we found no effect of the *cmeB* mutation on the ability of the bacteria to form this type of biofilm (Fig. 3A). Since C. jejuni motility plays an important role in the invasion of host cells (53), we also aimed to verify whether mutated bacteria remained motile. As shown in Fig. 3B, the *cmeB* mutation did not have any effect on bacterial motility.

### The CmeABC transporter and sequence variations of the CmeB protein contribute to tetracycline resistance.

The tetracycline resistance of some Campylobacter strains is associated with the *tetO* gene carried by a pTet plasmid (6). In a previous study, we observed that C. jejuni strain G1, which was isolated from a patient with GBS, was more resistant to tetracycline than was the 11168H strain, despite the absence of the *tetO* gene in both strains (54). Comparison of the genome of the G1 strain with that of reference strain NCTC 11168 revealed a remarkable difference in the sequences of the *cmeB* genes (54). The protein sequence identity for *cmeB* genes from these two strains was only 81%, while that for genes from strains 11168H and 81-176 was as high as 99%. In this study, we found that inactivation of *cmeB* in the G1 strain resulted in greater reduction of resistance to tetracycline than did inactivation of *cmeB* in the 11168H strain (Table 1). However, transfer of the pTet plasmid from C. jejuni 81-176 to the G1 strain by conjugation made the latter 8 times more resistant to tetracycline than the donor C. jejuni 81-176 carrying this plasmid (Table 1). This finding suggests that the CmeABC MDR efflux pump of C. jejuni strain G1 has greater ability to excrete this drug than do those of C. jejuni strains 11168H and 81-176. Since the nucleotide sequence differences were predominantly limited to *cmeB*, the variation in the efficiency of these pumps may be primarily associated with the product of this gene (Tables S1 and S2).

In addition, it was found that C. jejuni strain G1 was more resistant than strain 81-176 to various antibiotics and that the disruption of *cmeB* (confirmed with three clonal isolates) increased the susceptibility of the G1 strain to almost all antibiotics tested. The exceptions were penicillin, linezolid, and trimethoprim, for which no difference could be found due to very low (undetectable) levels of inhibition, probably due to low concentrations of the antibiotics used (Fig. S4). These data show that the spectrum and level of antimicrobial resistance of C. jejuni may be primarily dependent on the sequence variations of *cmeB*, with the potential for certain mutations in this gene to result in “super” efflux pump variants, significantly enhancing bacterial resistance to multiple antibiotics.

## DISCUSSION

The association between free-living amoebae and pathogenic bacteria is concerning, because it may have significant implications for human health (55). In this study, the interaction between C. jejuni 81-176 and A. polyphaga was investigated. C. jejuni displays extensive genetic variation; consequently, the efficiency by which C. jejuni interacts with cultured cells depends on specific properties of the strains (2, 56).

This study suggests that, during longer periods of incubation with A. polyphaga at 37°C, intracellular C. jejuni may escape into the extracellular medium, multiply, and reinfect amoebic cells. As the result, reinfection rather than intracellular multiplication may be the primary factor responsible for increased intracellular numbers. In order to obtain more accurate data about intracellular multiplication, an additional gentamicin treatment step was introduced in this study. Compared with the standard gentamicin assay, much more significant reductions in intracellular bacterial numbers were observed at both 25°C and 37°C. In the future, it would be interesting to investigate the interaction between C. jejuni and A. polyphaga at 42°C, as this is the chicken body temperature. It was observed previously that, at that temperature, the amoebic cells changed to the cyst shape (21). This is relevant, since amoebic cysts may play a role in contamination and the persistence of pathogenic bacteria in food-related environments, allowing internalized foodborne pathogens to resist the disinfection treatments used in the food industry (57). Despite gradual decreases in the numbers of intracellular bacteria at 25°C, the fact that intracellular bacteria are still detectable after 24 h might be of epidemiological importance if the amoebae are constantly exposed to the presence of these bacteria in the environment.

Our data also demonstrated prolonged extracellular survival of strain 81-176 in the presence of amoebae, which is in accordance with previously published results (41). According to Bui et al., this is likely to be due to the depletion of dissolved oxygen by amoebae, thus creating the microaerophilic environment optimal for C. jejuni growth (41).

At 37°C, the initial decrease in the number of viable bacteria was followed by a remarkable increase after 48 h of incubation. A similar trend was reported for S. enterica and Listeria monocytogenes (58). The authors termed the initial decrease in viability the eclipse phase, which probably occurs due to the initial use of the bacteria as a food source or due to a prolonged lag or adaptation phase, followed by active intracellular growth (58).

Although the CmeABC efflux pump of C. jejuni was reported previously to be required for colonization of the intestinal tract of chickens (5), its role in bacterium-host cell interactions was not determined. We were able to show that this pump is beneficial for the survival and replication of C. jejuni 81-176 within amoebae and that the reduction in the CFU numbers for the *cmeB* mutant was not the result of its greater sensitivity to the detergent used for amoebic lysis. These observations support the idea that efflux pumps may indeed act as virulence determinants. The role of MDR efflux pumps in virulence could be linked to their ability to expel and to confer resistance to host-derived antimicrobial agents, such as low-molecular-weight toxins (reactive oxygen species) and antimicrobial peptides (14).

**FIG 4:**
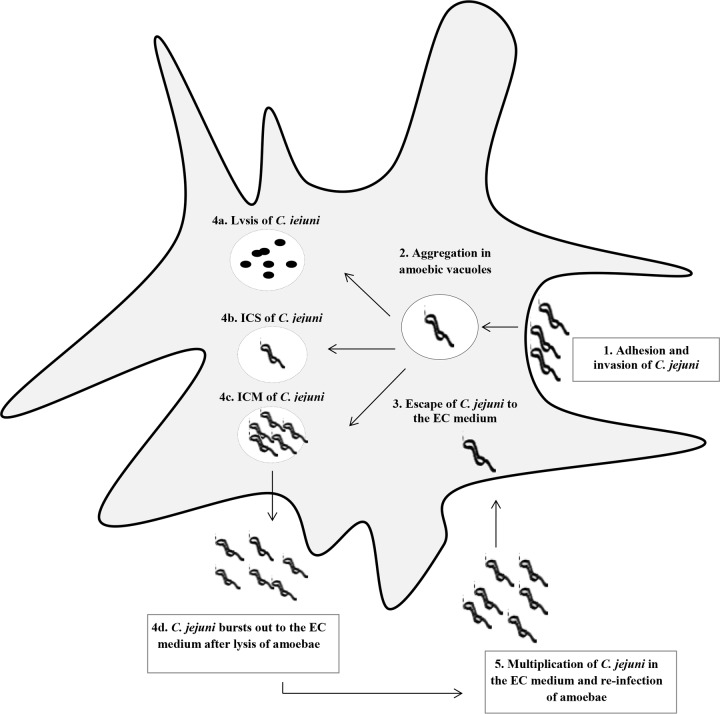
Hypothetical model of the interaction between C. jejuni and A. polyphaga. The following possible stages of bacterial entry are depicted: 1, adhesion to and invasion of amoebic cells via phagocytosis; 2, gathering within amoebic vacuoles (38, 40); 3, escape to the extracellular (EC) medium (39); 4a, bacterial cell lysis; 4b, intracellular survival (ICS) and escape without lysis; 4c, intracellular multiplication (ICM); 4d, escape after lysis followed by release into the extracellular medium (39); 5, presence in the extracellular medium. In the extracellular medium, C. jejuni is able to multiply and to reinfect other amoebic cells. Stages 1, 3, 4a to 4c, and 5 are based on the observations reported in this study.

Based on the information available in the literature and the data presented here, we suggest a hypothetical model describing a mechanism of interaction between C. jejuni and the amoebae (Fig. 4). According to this model, intracellular bacteria acquired from the environment (e.g., at 25°C, as in our experiments) multiply at 37°C (conditions simulating the host temperature). After ingestion of a product (e.g., water or milk) contaminated with amoebae, the latter are lysed, releasing large amounts of bacteria causing disease. A global search for other bacterial factors involved in the interaction between C. jejuni and amoebae could be based on differential expression studies (transcriptomics and proteomics). Our data also suggested that neither motility nor biofilm formation was responsible for the decreased survival of the *cmeB* mutant strain within the A. polyphaga host.

As demonstrated in this study, the exchange of genetic elements involved in antibiotic resistance can result in dramatic elevation of C. jejuni resistance to antibiotics. This is in accordance with other studies reporting C. jejuni CmeB sequence variants with much more powerful efflux of antibiotics, leading to enhanced antibiotic resistance (8, 59). The CmeB from C. jejuni strain G1 in this study shared 99% identity with the two super efflux pump variants discovered previously, i.e., the 154KU variant (8) and the RE-CmeB variant (59), suggesting that the CmeB from the G1 strain might also be a super efflux variant. However, this hypothesis needs to be supported by further studies. Despite much greater strain-to-strain sequence variation of CmeB, compared with other components of the MDR pump and CmeR, further experiments are required for evaluation of the relative contributions of these proteins to variations in the levels of antibiotic resistance. In this study, it was possible to generate bacteria with tetracycline resistance levels remarkably exceeding those of all parental strains. Such exchanges are likely to occur in the environment not only via conjugation (e.g., involving a transfer of the pTet plasmid) but also via transformation, as many strains of C. jejuni are naturally competent and can easily acquire DNA released due to the lysis of cells carrying antibiotic resistance genes (2). As a result, originally sensitive C. jejuni strains might easily become more resistant to antibiotics. Variation in CmeB structure is just one of various strategies utilized by bacteria for adaptation to hostile environmental conditions, both *in vivo* and *ex vivo*.

To summarize, we describe here the contribution of a Campylobacter efflux pump to bacterial survival within amoebae, which was demonstrated by employing an improved gentamicin protection method. Moreover, using a modified version of the gentamicin treatment method, the results explain the previously reported controversial results from investigations of Campylobacter-amoeba interactions. Because CmeABC is required for C. jejuni antibiotic resistance and virulence, this efflux pump is a promising target for interventions to combat C. jejuni infections.

## MATERIALS AND METHODS

### Bacterial strains and growth conditions.

C. jejuni strain 81-176 was used throughout this study because of its high virulence (60, 61) and enhanced ability to invade and to survive within A. polyphaga (according to our data). C. jejuni 11168H, a hypermotile variant of the reference strain NCTC 11168 (62), and G1, a strain isolated from a patient with Guillain-Barré syndrome (63), were used for the tetracycline resistance assay. Strain X was isolated from a patient with enteritis (49). C. jejuni strains were routinely grown for 24 h at 37°C on Columbia blood agar (CBA) (Oxoid) supplemented with 5% defibrinated horse blood and Campylobacter selective supplement (Skirrow; Oxoid), under microaerobic conditions (5% O_2_ and 10% CO_2_ in N_2_). NEB Express competent Escherichia coli used for molecular cloning was grown at 37°C in Luria-Bertani (LB) medium (Oxoid). When appropriate, the following antibiotics were included in the culture medium as selective agents: ampicillin (100 μg/ml), kanamycin (50 μg/ml), and tetracycline (10 μg/ml).

For liquid cultures, C. jejuni was suspended in brain heart infusion (BHI) broth (Oxoid) and adjusted to an optical density at 600 nm (OD_600_) of 1. The bacterial suspension was diluted 100-fold in BHI broth, in sterile conical flasks, and was incubated microaerobically at 37°C for 2 days, with shaking at 200 rpm. One-milliliter samples of each bacterial culture were taken at each time point (0, 6, 24, 30, and 48 h), and the OD_600_ was measured.

### Generation of C. jejuni 81-176 *cmeB* mutant and complemented strains.

Inactivation of the C. jejuni 81-176 gene *cmeB* was achieved by an insertional mutagenesis approach (49), in which *cmeB* was disrupted by insertion of a kanamycin resistance (*kan*^*r*^) cassette. The primers CmeB-F (5′-AAGGAGATATACCATGTTTTCTAAATTTTTTATAGAAAGACCTATTTTTG-3′) and CmeB-R (5′-TCATTCATGAATCTTACCTCTTTTTTTATCTAGC-3′) were used to amplify a 3-kb fragment containing the *cmeB* gene from C. jejuni 81-176 chromosomal DNA. The PCR product was ligated with pGEM-T Easy vector (Promega) using T4 DNA ligase (New England BioLabs), followed by transformation into NEB Express competent E. coli and selection of recombinant clones. The *kan*^*r*^ cassette was isolated from vector pJMK30 by digestion with SmaI (1.5 kb), followed by gel extraction of the 1.5-kb fragment containing the *kan*^*r*^ cassette. The plasmid pGEM-T Easy/*cmeB* was digested with ClaI and ligated with a DNA fragment containing the *kan*^*r*^ cassette by using T4 DNA ligase (Promega). After transformation of E. coli with the ligation mixture, the pGEM-T Easy/*cmeB*::*kan*^*r*^ plasmid containing the insert of interest, in the correct orientation, was isolated. The pGEM-T Easy/*cmeB*::*kan*^*r*^ plasmid was transformed into C. jejuni 81-176 via electroporation, and transformants were selected on CBA supplemented with kanamycin (50 μg/ml). The C. jejuni 81-176/*cmeB*::*kan*^*r*^ mutants were confirmed by PCR analysis.

The complemented strain C. jejuni 81-176/*cmeB*::*kan*^*r*^/*cmeB* was constructed via homologous recombination. The genomic DNA from the C. jejuni 81-176 strain was transformed into competent cells of the *cmeB* mutant strain via electroporation, and transformants were selected in CBA supplemented with tetracycline at 20 μg/ml, a concentration that does not support the growth of the *cmeB* mutant strain. A similar procedure was used for the construction of complementation derivatives of other strains (Table 1).

### Antibiotic susceptibility assays. (i) Broth microdilution assay.

The MICs were determined according to EUCAST guidelines (64). The MICs of tetracycline (Sigma) for C. jejuni strains were determined by a broth microdilution method, using Mueller-Hinton-fastidious (MH-F) broth (cation-adjusted Mueller-Hinton [MH] broth with 5% lysed blood and 20 mg/liter β-NAD). Briefly, 10 μl of C. jejuni suspension in MH-F broth (OD_600_ of 0.5) was added to 90 μl of 2-fold dilutions of tetracycline in MH-F broth. Suspensions were transferred to a 96-well flat-bottomed microtiter plate (Corning) and incubated for 3 days at 37°C under microaerobic conditions, with shaking at 100 rpm. The tetracycline concentration range tested was 0.03 μg/ml to 500 μg/ml, and control wells with no tetracycline were included. MICs were measured at 600 nm using an Infinite 200 PRO plate reader (Tecan). According to EUCAST guidelines, the tetracycline MIC breakpoints for C. jejuni are as follows: sensitive, ≤2 μg/μl; resistant, >2 μg/μl (64).

### (ii) Antibiotic disc diffusion assay.

The antibiotic susceptibility of C. jejuni strains was determined according to the EUCAST recommendations (64). C. jejuni was grown on CBA plates for 24 h at 37°C and suspended in 1 ml of MH broth at an OD_600_ of 0.5. The suspension (100 μl) was spread on MH agar plates supplemented with 5% lysed horse blood, using a spreader. Antibiotic-containing discs (Oxoid) were placed at the surface of the agar plates using sterile tweezers, and the plates were incubated for 2 days at 37°C under microaerobic conditions. Inhibition zone diameters were measured and interpreted accordingly to EUCAST zone diameter breakpoints for 30-μg tetracycline discs, as follows: sensitive, ≥30 mm; resistant, <30 mm (64).

### Amoebic culture conditions.

Acanthamoeba polyphaga (Linc Ap-1), which was used in all experiments, was kindly provided by Bernard de La Scola, University de La Mediterranee (Marseille, France). A. polyphaga was maintained aerobically at 25°C in peptone-yeast-glucose (PYG) medium (20 g protease peptone, 18 g glucose, 1 g yeast extract, 1 g MgSO_4_·7H_2_O, 1 g sodium citrate·2H_2_O, 0.02 g Fe(NH_4_)_2_(SO_4_)_2_·6H_2_O, 0.06 g CaCl_2_, 0.14 g H_2_PO_4_, and 0.35 g Na_2_HPO_4_·7H_2_O in 1 liter [pH 6.8]; filter sterilized with a 0.22-μm filter), in 75-cm^2^ treated culture flasks. The concentration and viability of amoebae were determined by the trypan blue exclusion assay, and amoebae were visualized by phase-contrast microscopy with a ×40 objective in an inverted cell culture microscope (Motic AE31).

To test the sensitivity of the C. jejuni 81-176 *cmeB* mutant to 0.1% (vol/vol) Triton X-100, bacterial cells were harvested from overnight CBA plates, suspended in BHI medium, and adjusted to an OD_600_ of 1 (initial inoculum). As controls, 100-μl aliquots of the initial inocula were serially diluted in phosphate-buffered saline (PBS) and plated on CBA plates for CFU counting. The bacterial suspensions were then centrifuged for 2 min at 10,000 × *g*, 1 ml of 0.1% (vol/vol) Triton X-100 was added, and the cells were incubated for 15 min at room temperature. Bacterial suspensions in Triton X-100 detergent were serially diluted in PBS and plated on CBA plates. The CFU counts obtained for the mutant strain were compared with those obtained for the WT and complemented strains.

### *In vitro* coculture assay.

Coculturing of C. jejuni with attached monolayers of A. polyphaga cells was conducted in 24-well plates. A. polyphaga cells were seeded at a density of 10^6^ amoebae per ml in PYG medium and were incubated at 25°C for 2 h to allow the cells to settle and to form monolayers at the bottom of the wells. Bacterial cells were harvested from overnight CBA plates, suspended in PYG medium, and adjusted to an OD_600_ of 1. Then, 100 μl of bacterial suspension was added to the wells with A. polyphaga, achieving multiplicities of infection ranging from 100 to 400 bacteria per well. To allow invasion to occur, cocultures were incubated under aerobic conditions for 2 h at 25°C or 37°C. Following coincubation, wells were washed once with PYG medium and treated for 1 h with 100 μg/ml gentamicin; this concentration was shown to be effective in fully eliminating the bacterial cells in 1 h (data not shown). Following gentamicin treatment, the wells were washed three times with PBS, and the amoebae were lysed with 0.1% (vol/vol) Triton X-100 for 15 min at room temperature, releasing the intracellular C. jejuni. Samples were serially diluted in PBS and plated on CBA plates, in duplicate, followed by 2 days of incubation at 37°C under microaerobic conditions. For longer incubations (24, 48, and 72 h), the wells were incubated with PYG medium without gentamicin. At the designated time points, prior to the addition of 0.1% Triton X-100, the wells were retreated with gentamicin (100 μg/ml) for 1 h (in a modification of the standard gentamicin protection assay). Cells were washed three additional times with PBS, lysed, diluted, and plated as described above. To calculate the number of intracellular bacteria, the following formula was used: [(recovered C. jejuni [in CFU per milliliter])/(total C. jejuni [in CFU per milliliter])] × 100 = % intracellular C. jejuni in A. polyphaga.

### Biofilm formation assay.

C. jejuni was grown on CBA plates at 37°C for 24 h, suspended in BHI broth, and adjusted to an OD_600_ of 0.5. The bacterial suspension (1 ml) was transferred to borosilicate glass tubes and incubated statically at 37°C for 4 days under microaerobic conditions, after which an attached pellicle at the gas-liquid interface was observed (52). For crystal violet (CV) staining, the glass tubes were washed twice with distilled water and dried at 85°C for 30 min. Then, 0.5% CV solution was added to the tubes, and the tubes were incubated at room temperature for 30 min, with gentle shaking. The tubes were washed with distilled water and dried at 85°C for 30 min. Lastly, 1 ml of a 80% ethanol-20% acetone mixture was added for 15 min, to dissolve the CV stain. Samples were transferred to 96-well plates in triplicate, and the OD_595_ was measured using an Infinite 200 PRO plate reader (Tecan).

### Motility assay.

The motility of C. jejuni was determined as described previously, with a few modifications (65). C. jejuni bacteria were grown on CBA plates at 37°C for 24 h, suspended in BHI broth, and adjusted to an OD_600_ of 0.5, after which 1-μl aliquots of the bacterial suspension were spotted onto 0.4% BHI soft agar plates. The low density of the agar allowed the bacteria to move within the agar, forming a halo of growth around the point of inoculation. Plates were incubated for 3 days at 37°C under microaerobic conditions.

### Conjugation of C. jejuni.

To generate the C. jejuni G1/pTet strain, the pTet plasmid from C. jejuni 81-176 was transferred to C. jejuni G1 by conjugation. A mixture of 200 μl of the 81-176 donor strain with 100 μl of the G1 recipient strain (MH medium inoculum at an OD_600_ of 1) was plated on top of a 0.22-μm filter membrane (Millipore), placed on the surface of a CBA plate, and incubated overnight at 37°C under microaerobic conditions. The bacterial growth was scraped from the membrane and plated on CBA supplemented with tetracycline (15 μg/ml) and ampicillin (5 μg/ml); the latter does not support the growth of the 81-176 strain but allows the G1 strain to grow. PCR was conducted to confirm the derivative strain using the primers pTet-F (5′-GGCGTTTTGTTTATGTGCG-3′) and pTet-R (5′-ATGGACAACCCGACAGAAGC-3′).

### Statistical analysis.

All experiments were repeated three times (biological replicates), with three technical replicates in each experiment, and the data were expressed as mean ± standard deviation (SD). Comparisons of two groups were made with an unpaired, two-tailed, Student's *t* test. Mean differences were considered statistically nonsignificant when the *P* values were >0.05.

## Supplementary Material

Supplemental material
