# Supplementary material for: CmeABC Multidrug Efflux Pump Contributes to Antibiotic Resistance and Promotes Campylobacter jejuni Survival and Multiplication in Acanthamoeba polyphaga
Source: Appl Environ Microbiol. 2017 Oct 31;83(22):e01600-17. doi: 10.1128/AEM.01600-17 (PMC5666138; doi:10.1128/AEM.01600-17)
Supplement: Supplemental material [file supp_83_22_e01600-17__index.html]

Supplemental material 

# The CmeABC Multidrug Efflux Pump Promotes Campylobacter jejuni Survival and Multiplication in Acanthamoeba polyphaga

## Supplemental material

- Supplemental file 1 -

  Comparison of sequences of genes *cmeA*, *cmeB*, *cmeC*, and *cmeR* and the respective proteins of *C. jejuni* strains G1 (query) and 11168H using NCBI BlastN and BlastP programs, respectively; comparison of sequences of genes *cmeA*, *cmeB*, *cmeC*, and *cmeR* and the respective proteins of *C. jejuni* strains G1 (query) and 81-176 using NCBI BlastN and BlastP programs, respectively (Table S2); extracellular survival of *C. jejuni* 81-176 in the presence of *A. polyphaga* (Fig. S1); growth of *C. jejuni* 81-176 is not affected by the *cmeB* mutation (Fig. S2); susceptibility of *C. jejuni* wild-type, *cmeB* mutant, and complemented strains to Triton X-100 (Fig. S3); antibiotic disk diffusion assay of *C. jejuni* strains (Fig. S4); quantification of *C. jejuni* bacteria within *A. polyphaga* (Fig. S5).

  PDF, 490K
