# Supplementary material for: CmeABC Multidrug Efflux Pump Contributes to Antibiotic Resistance and Promotes Campylobacter jejuni Survival and Multiplication in Acanthamoeba polyphaga
Source: Appl Environ Microbiol. 2017 Oct 31;83(22):e01600-17. doi: 10.1128/AEM.01600-17 (PMC5666138; doi:10.1128/AEM.01600-17)
Supplement: Supplemental material [file AEM.01600-17_zam999118154s1.pdf]

G1 vs 11168H

	Amino acid sequence identity (coverage)	Nucleotide sequence identity (coverage)
**CmeA**	97% (100%)	96% (100%)
**CmeB**	81% (100%)	80% (99%)
**CmeC**	99% (98%)	98% (100%)
**CmeR**	94% (100%)	93% (100%)

Table S1. Comparison of sequences of genes *cmeA*, *cmeB*, *cmeC* and *cmeR* and respective proteins of *C. jejuni* strains G1 (query) and 11168H using NCBI BlastN and BlastP programs respectively.

G1 vs 81-176

	Amino acid sequence identity (coverage)	Nucleotide sequence identity (coverage)
**CmeA**	97% (100%)	96% (100%)
**CmeB**	82% (100%)	80% (100%)
**CmeC**	99% (98%)	98% (100%)
**CmeR**	95% (100%)	94% (100%)

Table S2. Comparison of sequences of genes *cmeA*, *cmeB*, *cmeC* and *cmeR* and respective proteins of *C. jejuni* strains G1 (query) and 81-176 using NCBI BlastN and BlastP programs respectively.

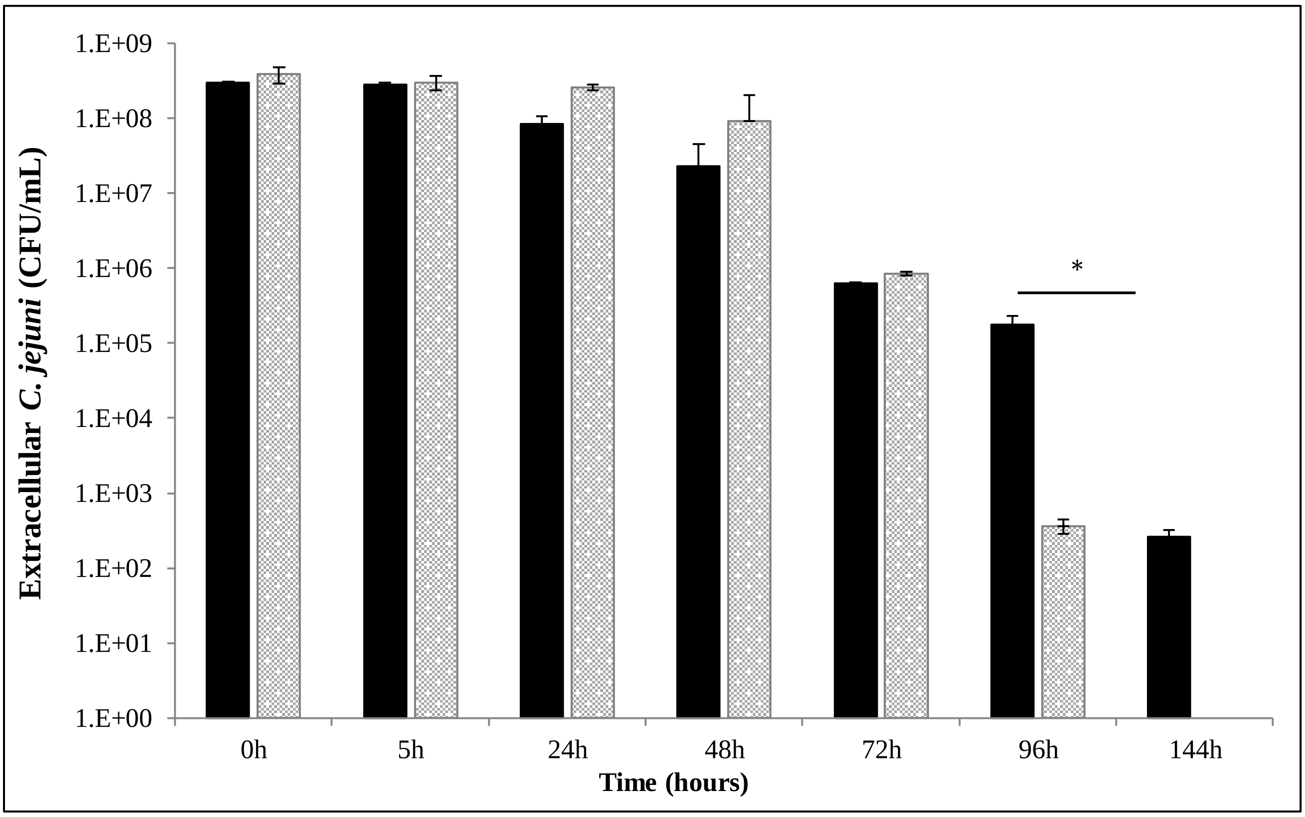

Figure S1. Extracellular survival of *C. jejuni* 81-176 in the presence of *A. polyphaga*. Extracellular survival was determined by cfu counting at 0, 5, 24, 48, 72, 96 and 144 hours post-infection at 25°C in aerobic conditions. Black bars represent bacterial cfu counts for strain 81-176 wt in co-culture with *A. polyphaga* and grey bars correspond to when in PYG medium alone. For 96h p= 0.044.

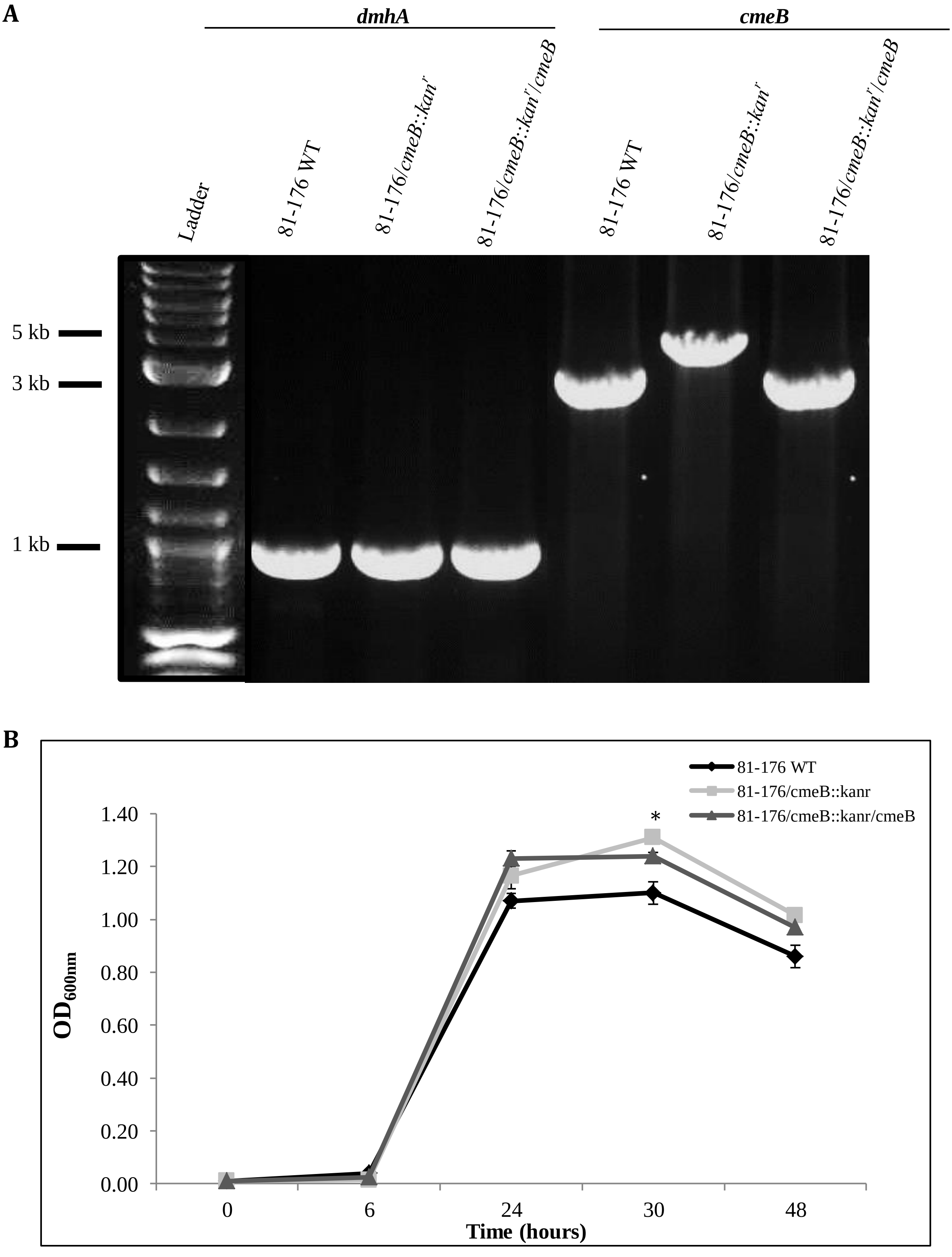

Figure S2. Growth of *C. jejuni* 81-176 is not affected by *cmeB* mutation (A) PCR results: Lane L: 2-log DNA ladder; Lane 1: 81-176 – 1021 bp; Lane 2: 81-176/*cmeB::kan*^{r} – 1021 bp; Lane 3: 81-176/*cmeB::kan*^{r}/*cmeB* – 1021 bp; Lane 4: 81-176 – 3.1 kb; Lane 5: 81-176/*cmeB::kan*^{r} – 4.6 kb; Lane 6: 81-176/*cmeB::kan*^{r}/*cmeB* – 3.1 kb. Increased fragment size with the latter indicates insertion of the *kan*^{R} cassette and consequently, disruption of the *cmeB* gene. (B) Growth rates of 81-176 wt (black), 81-176/*cmeB::kan*^{r} mutant (light grey) and 81-176/*cmeB::kan*^{r}/*cmeB* derivative (dark grey).

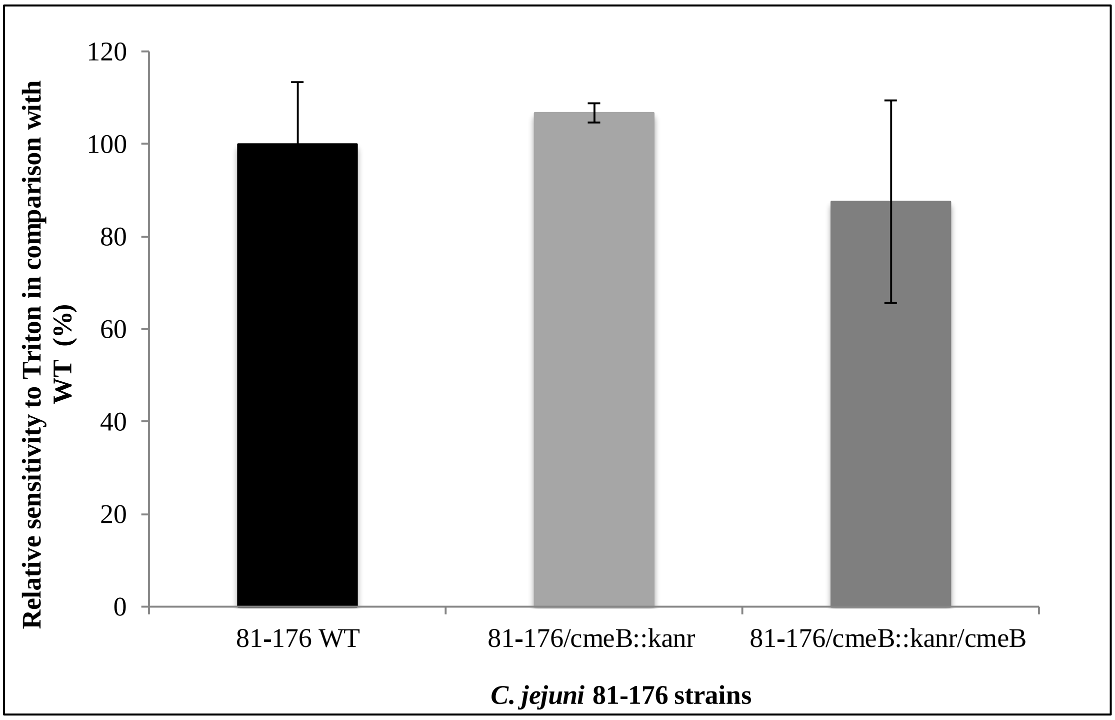

Figure S3. Susceptibility of *C. jejuni* wt, *cmeB* mutant and complement strains to Triton X-100. Sensitivity to Triton was determined by cfu counts after 15 minutes exposure to Triton X-100 0.1% (v/v) in aerobic conditions. Colour coding: black, 81-176 wt strain; light grey, 81-176/*cmeB*::kan^r mutant strain; dark grey, 81-176/*cmeB*::kan^r /*cmeB* complementation strain.

A

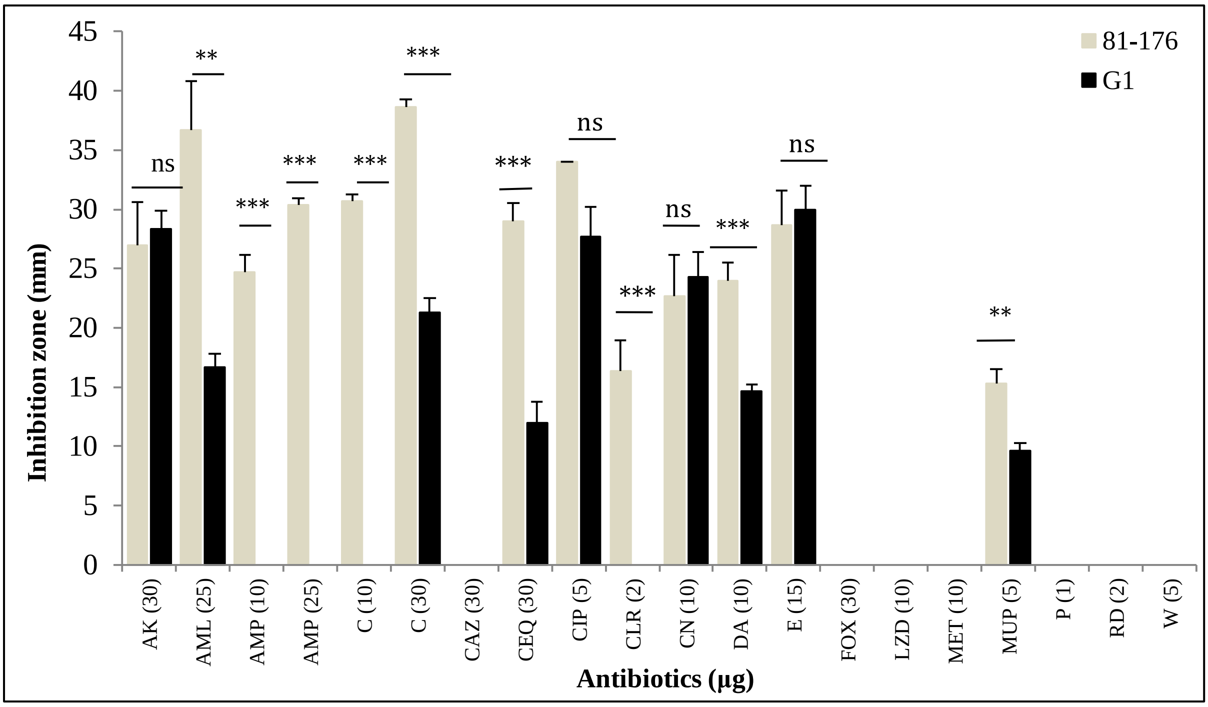

B

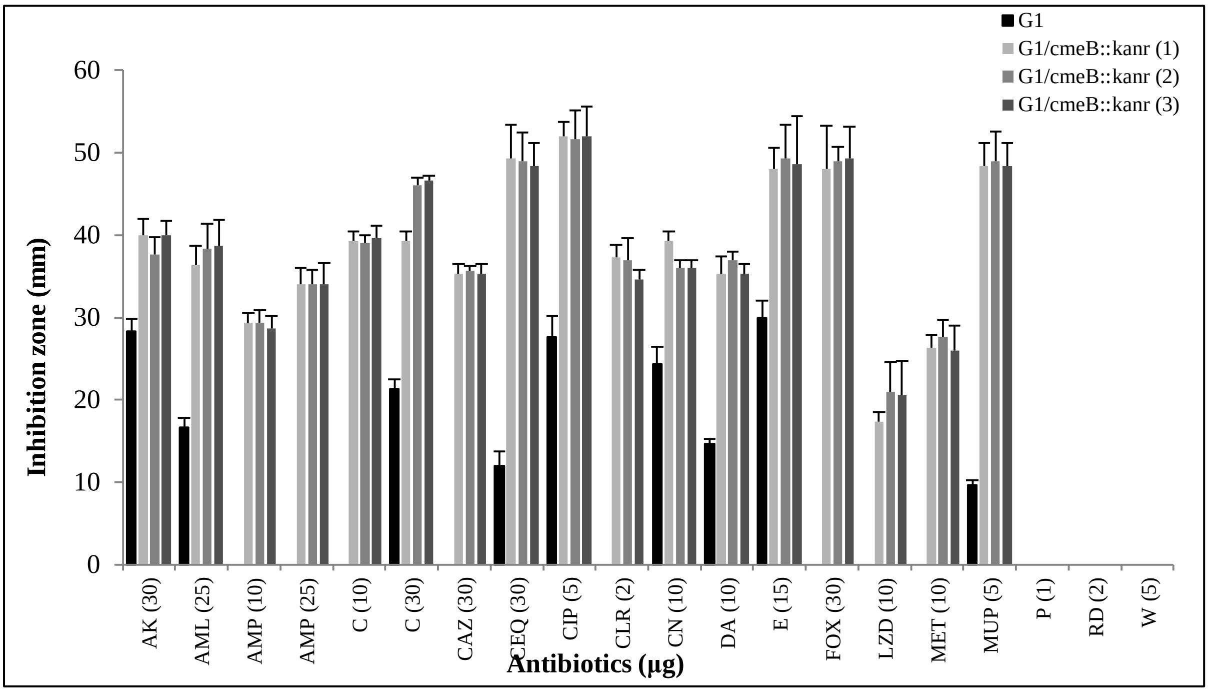

Figure S4. Antibiotic disc diffusion assay to *C. jejuni* strains. The susceptibility of (A) *C. jejuni* G1 (black bars) and 81-176 (beige bars) and (B) G1/*cmeB*::*kan*^{r} mutant strains (grey bars) to different types of antibiotics was determined by the disc diffusion assay in MH blood agar plates, according to EUCAST recommendations. Absence of bars indicates full resistance of the *C. jejuni* strains. Amikacyn (AK), amoxicillin (AML), ampicillin (AMP), chloramphenicol (C), ceftazidime (CAZ), cefquinome (CEQ), ciprofloxacin (CIP); clarithromycin (CLR), gentamycin (CN), clindamycin (DA), erythromycin (E), cefoxitin (FOX), linezolid (LZD), methicillin (MET), mupirocin (MUP), penicillin G (P), rifampicin (RD) and trimethoprim (W). The antibiotic concentration used (µg/ml) is represented in brackets. Three clonal isolates of G1/*cmeB*::*kan*^{r} mutants were tested. Statistically significant differences with p value < 0.001 were obtained for all the antibiotics tested between the G1 WT strain and the three G1/*cmeB*::*kan*^{r} clonal isolates.

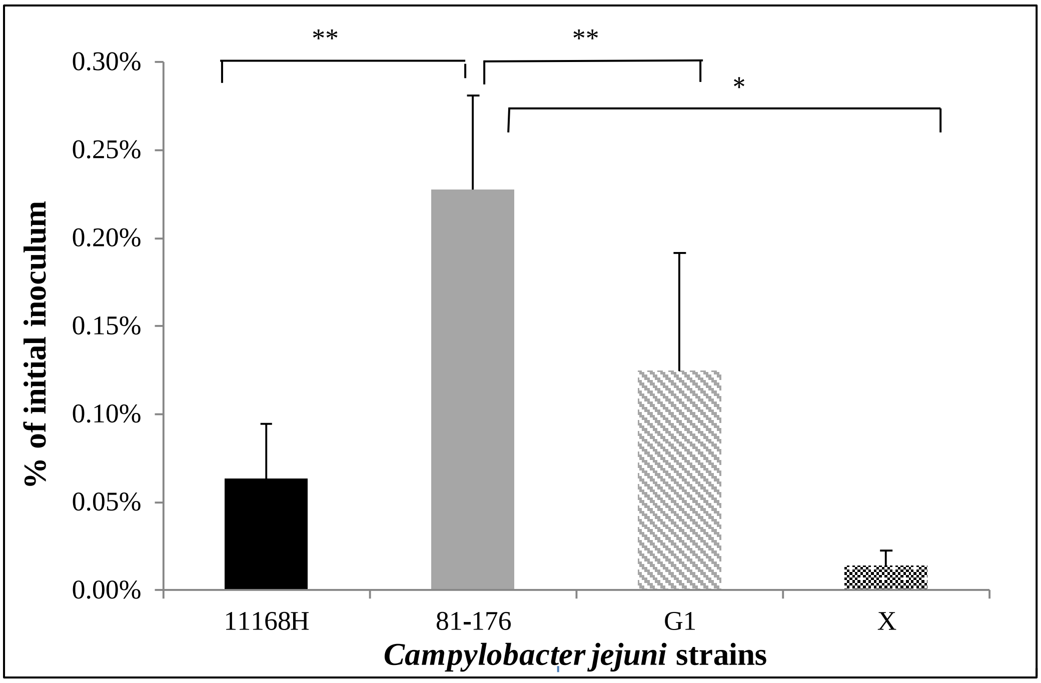

Figure S5. **Quantification of *C. jejuni* within *A. polyphaga*.** Quantification of intracellular bacteria was determined by c.f.u counts at 0h post-gentamicin treatment at 25°C in aerobic conditions. Values are mean ± SD from 3 independent experiments. Statistically significant differences for 11168H, G1 and X were obtained in comparison with strain 81-176 (p=0.003; p=0.007; p=0.0016, respectively).
